# Supplementary material for: Inconsistent effects of components as evidence for non-compositionality in chimpanzee face-gesture combinations? A response to Oña et al (2019)
Source: PeerJ. 2024 Feb 21;12:e16800. doi: 10.7717/peerj.16800 (PMC10893859; doi:10.7717/peerj.16800)
Supplement: Supplemental Information 1 — ‘Model’ describes the dependent variable (left of the tilde ‘~’) and the fixed effects (right of the tilde ‘~’, “1” means intercept only). ‘Filters’ describe the subset of the data on which the model was fitted and ‘Estimates’ report the estimates and p-values of the intercept and regression coefficient when suited. All details (e.g., random structure) are available in the R script at https://osf.io/gny5k. [file peerj-12-16800-s001.docx]

| Model | Filters | | | Estimates (and *p*-values) | | | |
| --- | --- | --- | --- | --- | --- | --- | --- |
|  | Faces | Recipient rank | Gestures | Intercept | | β | |
| score   ~ rank | Neutral only | all | all | 1.05 | (*p*=0.11) | 1.37 | (*p*=0.01*) |
| score   ~ 1 | Neutral only | Dominant only | BG only | 0.03 | (*p*=0.98) | – |  |
| score  ~ 1 | Neutral only | Dominant  only | SG only | -11.13 | (*p*=0.02*) | – |  |
| gesture  ~ rank | Neutral  only | all | all | 0.97 | (*p*=0.04*) | 1.17 | (*p*=0.07 .) |
| score   ~ face | Neutral vs Hoot | all | all | 1.09 | (*p*=0.22) | -1.15 | (*p*=0.03*) |
| score  ~ face | Neutral vs Bared | Dominant only | BG only | 0.01 | (*p*=0.99) | -2.18 | (*p*=0.09 .) |
| score  ~ face | Neutral vs Bared | Dominant only | SG only | -10.84 | (*p*=0.01*) | 22.63 | (*p*=0.01*) |
